# Acute IL-4 Governs Pathogenic T Cell Responses during *Leishmania major* Infection

**DOI:** 10.4049/immunohorizons.2000076

**Published:** 2020-09-18

**Authors:** Barun Poudel, Matthew S. Yorek, Lalita Mazgaeen, Scott A. Brown, Thirumala-Devi Kanneganti, Prajwal Gurung

**Affiliations:** * Iowa Inflammation Program, University of Iowa, Iowa City, IA 52242;; † Department of Internal Medicine, University of Iowa, Iowa City, IA 52242;; ‡ Interdisciplinary Graduate Program in Human Toxicology, University of Iowa, Iowa City, IA 52242;; § Department of Immunology, St. Jude Children’s Research Hospital, Memphis, TN 38105;; ¶ Immunology Graduate Program, University of Iowa, Iowa City, IA 52242;; ‖ Center for Immunology and Immune Based Disease, University of Iowa, Iowa City, IA 52242

## Abstract

*Leishmania* spp. infection is a global health problem affecting more than 2 million people every year with 300 million at risk worldwide. It is well established that a dominant Th1 response (IFN-γ, a hallmark Th1 cytokine) provides resistance, whereas a dominant Th2 response (IL-4, a hallmark Th2 cytokine) confers susceptibility during infection. Given the important role of IL-4 during *L. major* infection, we used IL-4–neutralizing Abs to investigate the cellular and molecular events regulated by IL-4 signaling. As previously published, neutralization of IL-4 in *L. major*–infected BALB/c mice (a *Leishmania* susceptible strain) provided protection when compared with control *L. major*–infected BALB/c mice. Despite this protection, IFN-γ production by T cells was dramatically reduced. Temporal neutralization of IL-4 revealed that acute IL-4 produced within the first days of infection is critical for not only programming IL-4–producing Th2 CD4^+^ T cells, but for promoting IFN-γ produced by CD8^+^ T cells. Mechanistically, IL-4 signaling enhances anti-CD3–induced Tbet and IFN-γ expression in both CD4^+^ and CD8^+^ T cells. Given the pathogenic role of IFN-γ–producing CD8^+^ T cells, our data suggest that IL-4 promotes cutaneous leishmaniasis pathology by not only promoting Th2 immune responses but also pathogenic CD8^+^ T cell responses. Our studies open new research grounds to investigate the unsuspected role of IL-4 in regulating both Th1 and Th2 responses. *ImmunoHorizons*, 2020, 4: 546–560.

## INTRODUCTION

*Leishmania* spp. infection has been used as a major immunological tool, leading to the discovery of Th1 and Th2 immune responses (1). Studies conducted with *Leishmania major* in different strains of mice led to the discovery of Th1 and Th2 immunity (2). *L. major* infection of resistant mouse strains that include C57BL/6, positively correlate with a predominant Th1 response hallmarked by IFN-γ (3). Mechanistic studies have identified IL-12 as the innate cytokine critically required for programming a Th1-predominant response and resistance to *L. major* infection in these resistant mice (4, 5). Conversely, *L. major* infection of susceptible mouse strains, including BALB/c mice, elicit a Th2-dominant immune response hallmarked by IL-4 cytokines (6, 7). The importance of IFN-γ and IL-4 cytokines in regulating anti-leishmanial immunity has been extensively studied in vitro and in vivo.

Addition of rIFN-γ to *Leishmania* spp.–infected macrophages accelerates parasite clearance in vitro (8, 9). Specifically, IFN-γ produced by T cells activates macrophages to kill the intracellular *L. major* parasites (8, 10). To directly test the role of IFN-γ in vivo, the course of *L. major* infection was examined in IFN-γ–deficient mice on a resistant C57BL/6 background (11). Although C57BL/6 mice resolve the *L. major* infection over time, IFN-γ–deficient mice not only fail to resolve but develop fatal *L. major* infection. In concurrence, IFN-γR–deficient mice on a resistant 129 background failed to resolve *L. major* infection (12). In support of the genetic data, neutralization of IFN-γ during *L. major* infection of resistant mouse strains (129 and C57BL/6) also promotes exacerbated lesions that fail to resolve (13, 14). Cellular analysis has revealed that IFN-γ–deficient mice (genetic or neutralization) had a predominant Th2 response as demonstrated by increased levels of IL-4–producing CD4^+^ T cells and IL-4 cytokines measured in the lesions (11, 14). Altogether, these studies demonstrate a critical protective role for IFN-γ–producing Th1 CD4^+^ T cells during *L. major* infection. Interestingly, more recent work by Carneiro et al. (15) suggests that early IFN-γ production is required for recruitment of parasite-permissive monocytes, suggesting early Th2 responses that cross-regulate Th1 responses (i.e., IFN-γ) may be beneficial for the host.

Unlike IFN-γ, the role of IL-4 during *L. major* infection has been contested and remains unresolved to some extent. Early studies investigating the role of IL-4 in susceptible BALB/c mice used neutralizing Abs to deplete IL-4 in BALB/c mice during *L. major* infection. As expected, IL-4 neutralization provided significant protection from *L. major*–induced cutaneous lesions and swelling in susceptible BALB/c mice (14, 16, 17). Importantly, neutralization of IL-4 produced at the time of infection was sufficient to provide protection in susceptible BALB/c hosts (17). Moreover, IL-4 transgenic mice on a 129 background (genetically resistant mice that express IL-4 under control of the IgG H chain) are highly susceptible to *L. major* infection (18). In contrast, addition of rIL-4 in BALB/c mice during *L. major* infection did not worsen the disease (19). Indeed, rIL-4 treatment promoted a dominant Th1 response and clearance of lesion pathology during *L. major* infection of BALB/c mice (19, 20).

In support of IL-4 in promoting Th1 responses, several other groups have found that IL-4 can indeed promote IFN-γ production by CD4^+^ and CD8^+^ T cells during various stimulatory conditions (21–25). Similar disagreements remain with studies in mice genetically deficient in IL-4 within the BALB/c background. One group showed that IL-4–deficient mice generated on a 129 background and backcrossed to a BALB/c background for six generations were resistant to *L. major* infection when compared with BALB/c controls (26). Given the resistant background of the IL-4–deficient 129 mice to which BALB/c mice were backcrossed, it could be argued that the incomplete backcrossing could account for the resistance observed in IL-4–deficient mice. However, the same authors also tested IL-4–deficient mice generated with BALB/c mice, which were also resistant to *L. major* infection when compared with BALB/c controls (26). In support of a pathogenic role for IL-4, IL-4Ra–deficient mice (deficient in both IL-4 and IL-13) in a BALB/c background are highly resistant to *L. major* infection as well (27, 28). Interestingly, other studies have not found any difference in BALB/c mice versus IL-4–deficient mice (generated from BALB/c embryonic stem cells) during *L. major* infection (29, 30). Thus, it remains unclear how IL-4 modulates immune responses during *L. major* infection in BALB/c mice and why there is disagreement between studies about the role of IL-4 between different groups.

One point that has been undisputed in the literature is that IL-4 neutralization (via anti–IL-4 Ab) at early time points following *L. major* infection provides significant protection from disease in BALB/c mice (16, 31). Thus, it is possible that IL-4 could have different roles temporally during *L. major* infection. In this study, we have used temporal neutralization of IL-4 in BALB/c mice during *L. major* infection to examine the overall outcome on the disease and immune response. Using IL-4 neutralization in BALB/c mice during *L. major* infection, we have not only confirmed previous studies, but provide data to support the dual role for IL-4 in promoting both Th2 and Th1 responses. In brief, we show that early IL-4 is critical for the development of IL-4–producing CD4^+^ Th2 cells, but more strikingly, this acute IL-4 is also critical for optimal IFN-γ production by CD4^+^ and CD8^+^ T cells. Given recent studies demonstrating a pathogenic role for CD8^+^ T cells during *L. major* infection (32, 33), our study shows that IL-4–induced pathology during *L. major* infection may be 2-fold: first, IL-4 supports a Th2 immune response highlighted by IL-4–producing CD4^+^ T cells, and second, IL-4 promotes a pathogenic IFN-γ producing CD8^+^ T cells. Mechanistically, we show that IL-4 directly enhancesboth Th2-associatedGATA3 and Th1-associated Tbet transcription factors in CD4^+^ and CD8^+^ T cells to drive IL-4 and IFN-γ cytokine expression in these cells. In conclusion, our studies demonstrate a previously unappreciated role for IL-4 in optimal priming and activation of CD4^+^ and CD8^+^ T cell responses that may contribute to the observed pathology in BALB/c mice during *L. major* infection.

## MATERIALS AND METHODS

### Mice

Six- to 12-wk-old BALB/cJ mice (stock number 000651) were purchased from The Jackson Laboratory and housed and bred at a specific pathogen–free facility at the University of Iowa. Experimental procedures were approved by the University of Iowa Animal Care and Use Committee (approved Animal Protocol 0032004) and performed in accordance with the Office of Laboratory Animal Welfare guidelines and the Public Health Service Policy on Humane Care and Use of Laboratory Animals.

### L. major *infections*

The *L. major* strain WHOM/IR/−173 was grown in vitro in complete M199 media supplemented with 5% HEPES, 10% FBS, and 1% penicillin–streptomycin at 27°C as described previously (34). One million metacyclic promastigotes of *L. major* were injected per footpad in a volume of 50 μl. Footpad measurements were taken weekly using a caliper. For immune cell phenotyping, popliteal lymph nodes (PLN) were recovered at different timepoints and single-cell suspensions were prepared. For quantification of *L. major* promastigotes, footpads, spleen, and PLN were homogenized, and serial limiting dilutions of the homogenates were plated in 96-well flat-bottom plates in complete M199 media. Five to six days after culture, each well was analyzed under a microscope for the presence or absence of *L. major* to determine the titers.

### Human PBMC isolation and culture

PBMCs were isolated from blood collected from healthy volunteers, as described previously (35). Written consents were obtained from each volunteer in accordance with a protocol approved by the Institutional Review Board for Human Subjects at the University of Iowa. Briefly, heparinized blood was subjected to dextran sedimentation followed by density gradient centrifugation in Ficoll-Paque PLUS (GE Healthcare, Chicago, IL) to isolate the PBMCs. Cells were cultured in complete media containing RPMI 1640 (Life Technologies, Waltham, MA), 10% heat-inactivated FBS (Life Technologies), and 1% penicillin–streptomycin (Invitrogen, Carlsbad, CA). PBMC were cultured in the presence or absence of 20 ng/ml human rIL-4 (catalog no. RCP05112; Reprokine, Valley Cottage, NY) with or without 1 mg/ml anti-CD3 (OKT3; Tonbo Biosciences, San Diego, CA) for the indicated timepoints and subjected to flow cytometry analysis as described below.

### Splenocyte isolation and culture

Spleens were harvested from mice, erythrocytes were depleted using ammonium-chloride-potassium lysis buffer, and single-cell suspension was prepared. The cells were counted, and 0.2 million cells were cultured in complete media and stimulated with or without 1 μg/ml anti-CD3 (17A2; Tonbo Biosciences) in the presence or absence of 20 ng/ml rIL-4 (catalog no. RKP07750; Reprokine) for the indicated times. For intracellular cytoplasmic staining (ICS), spleen cells were cultured in the presence of 1× brefeldin A (BioLegend, San Diego, CA) for 4–6 h. For transcription factors, splenocytes were cultured for 24 h followed by intranuclear staining. After incubation for the desired timepoints under nonstimulaotry or stimulatory conditions, cells were harvested for flow cytometry analysis as described below.

### CD4^+^ and CD8^+^ T cell isolation and culture

CD4^+^ and CD8^+^ T cells were isolated from spleen by negative magnetic selection using Dynabeads Untouched Mouse CD4 (catalog no. 11415D; Thermo Fisher Scientific, Waltham, MA) or CD8 Cells Kit (catalog no. 11417D; Thermo Fisher Scientific), according to the manufacturer’s instructions. The purity of the cells obtained was (>90%). Following isolation, purified CD4^+^ and CD8^+^ T cells were stimulated with anti-CD3 in the presence or absence of rIL-4. In some experiments, purified CD8^+^ T cells were stimulated with 10 ng/ml PMA and 500 nM ionomycin for 4 h in the presence of brefeldin A. For ICS, purified cells were cultured in the presence of 1× brefeldin A for 4–6 h. For transcription factors, splenocytes were cultured for 24 h followed by intranuclear staining. At the end of the stimulation, purified T cells were prepared for flow cytometry analysis.

### Flow cytometry

Surface staining was carried out using PBS with 1% FBS. For intracellular staining of cytokines, cells were first surface stained, then fixed with 1% paraformaldehyde (catalog no. 19943; Affymetrix, Santa Clara, CA) for 15 min at room temperature and permeabilized in permeabilization buffer (TNB-1213-L150; Tonbo Biosciences) before staining for cytokines. Staining for transcription factors was performed with the Foxp3/Transcription Factor Staining Buffer Kit (TNB-1022-L160; Tonbo Biosciences) according to the manufacturer’s instructions. Flow cytometry data were collected with CytoFLEX (BD Biosciences, Franklin Lake, NJ), and results were analyzed using FlowJo software (Becton Dickinson, Franklin Lakes, NJ). Abs specific for mouse CD4 (RM4–5), CD8 (53–6.7), and IL-4 (11B11) were purchased from Tonbo Biosciences; and those for IFN-γ (4S.B3), TNF (MAB11), GATA3 (L50–823), and Tbet (eBio4B10) were purchased from eBioscience (San Diego, CA). Abs specific for human CD4 (RPA-T4), CD8 (RPA-T8), and IL-4 (MP4–25D2) were purchased from Tonbo Biosciences; and those for IFN-γ (4S.B3), GATA3 (16E10A23), and Tbet (eBio4B10) were obtained from eBioscience.

### IL-4 neutralization

To neutralize IL-4 in vivo, mice were administered 400 μg of IL-4 Ab in 200 μl of PBS (11B11) i.p. on the days indicated. Control mice were administered anti-mouse IgG (catalog no. 0855455; MP Biomedicals, Santa Ana, CA) or PBS as control.

### Cytokine analysis by ELISA

Footpads homogenized in 1 ml of PBS with 1× complete protease inhibitor mixture (catalog no. 11697498001; Sigma-Aldrich, St. Louis, MO) were subjected to centrifugation, and clear supernatant was analyzed for the indicated cytokines. For in vitro experiments, PLN cell culture supernatants were collected at the end of the experiment and processed for the detection of indicated cytokines using ProcartaPlex ELISA kits for IL-4 and IFN-γ (catalog no. EPX010-20440-901; Thermo Fisher Scientific).

### Statistical analyses

Statistical analysis was performed and figures were generated using GraphPad Prism 8.0 software (GraphPad Software, San Diego, CA). Statistical significance was determined using either *t* tests (two-tailed and Mann–Whitney) for two groups or one-way ANOVA (with Dunnett or Tukey multiple comparisons tests) for three or more groups. All values are expressed as mean 6 SEM. For survival curve analysis, a log-rank (Mantel–Cox) test was used. A *p* value <0.05 was considered statistically significant.

## RESULTS

### *Chronic IL-4 neutralization during* L. major *infection provides protection from nonhealing lesions in BALB/c mice*

**FIGURE 1.**
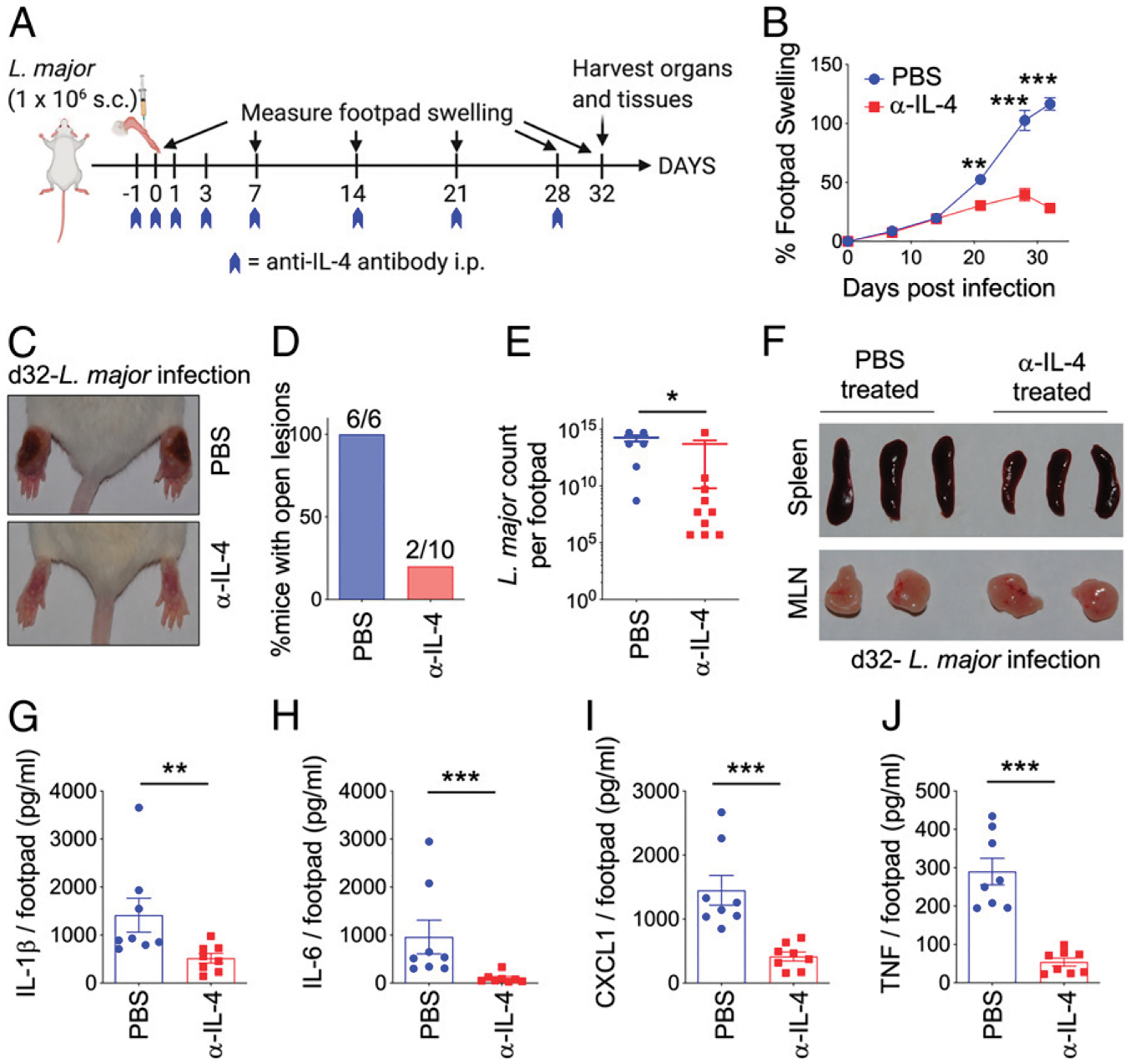
Chronic IL-4 neutralization protects susceptible BALB/C mice from *L. major* infection. (**A**) Schematic diagram of experimental design. Mice were infected in the footpads s.c. with one million *L. major* metacyclic promastigotes in 50 μl volume. On days −1, 0, 1, 3, 7, 14, 21, and 28 postinfection, mice were injected i.p. with PBS or 400 μg of anti–IL-4 Ab. Footpad measurements were taken once weekly, and mice were euthanized on day 32 postinfection. (**B**) Measurement of footpad swelling in PBS- and anti–IL-4–treated BALB/c mice following *L. major* infection. (**C**) Representative images of footpads of PBS- and anti–IL-4–treated *L. major*–infected mice. (**D**) Graphical representation of mice with lesions in PBS- and anti–IL-4–treated *L. major*–infected mice. (**E**) On day 32 postinfection, footpads were homogenized to determine *L. major* titers using limiting dilution assay. (**F**) Representative images of spleens and PLN from PBS- and anti–IL-4–treated *L. major*–infected mice. (**G**–**J**) IL-1β, IL-6, CXCL1, and TNF cytokine levels were determined by multiplex ELISA in footpad lysates. Each dot represents an individual mouse. Data represent the mean ± SEM. *p < 0.05, **p < 0.01, ***p < 0.001.

Previous studies using IL-4 neutralization (14, 17, 31, 36), rIL-4/IL-4 transgenic (18, 19), or IL-4–deficient mice (26, 28, 29, 37) in a BALB/c background have presented conflicting results on the role of IL-4 during *L. major* infection. We posited that possible temporal effects of IL-4 during different stages of *L. major* infection may explain the discrepant conclusions observed in previous studies. To this end, we started our experiment by performing a complete IL-4 neutralization using mouse anti–IL-4 mAbs (Clone 11B11). Specifically, we treated mice with 400 μg of anti–IL-4 Ab on days −1, 0, 1, 3, 7, 14, 21, and 28 post-*L. major* infection (Fig. 1A). Weekly, measurement of footpad swelling showed that anti–IL-4–treated groups were significantly protected from footpad swelling over the course of 32 d (Fig. 1B). Representative footpad images show significant footpad swelling and lesion in PBS-treated BALB/c mice that are absent in anti–IL-4–treated groups (Fig. 1C). Although 100% of PBS-treated groups had lesions with open wounds, only 20% of anti–IL-4–treated groups presented with open wound lesions (Fig. 1D). This lesion pathology was associated with significantly reduced *L. major* burden in the footpad, although it should be noted that the anti–IL-4–treated mice still had a significant *L. major* count despite reduced footpad swelling and lesion pathology (Fig. 1E). Representative images of spleen and draining PLN from PBS- or anti–IL-4–treated BALB/c mice following *L. major* infection did not show any differences (Fig. 1F). As expected, proinflammatory cytokines IL-1β, IL-6, CXCL1, and TNF were also significantly reduced in the footpads of anti–IL-4–treated *L. major*–infected BALB/c mice (Fig. 1G–J). Altogether, these results show that chronic IL-4 neutralization provides significant protection from *L. major* infection in BALB/c mice.

We next examined the immune composition of the PLN to determine any cellular differences in PBS versus anti–IL-4–treated BALB/c mice during *L. major* infection. CD4^+^ T cells determined by CD4 and TCR-β expression were present in similar frequency and numbers in PLN of PBS-treated mice when compared with anti–IL-4–treated groups (Supplemental Fig. 1A). Previous studies have shown that CD11a expression on CD8^+^ and CD4^+^ T cells can be used as a surrogate marker to track activated T cells (38, 39). There were no differences in frequency and total numbers of CD4^+^CD11a^hi^ T cells in PLN between PBS- and IL-4–treated groups (Supplemental Fig. 1B). CD8^+^ T cell frequency, as determined by CD8 and TCR-β expression, was significantly reduced in anti–IL-4–treated groups, but the numbers were similar (Supplemental Fig. 1C). In contrast to CD4^+^ T cells, we observeda significant reduction in frequency and total numbers of CD8^+^ CD11a^hi^ cells in anti–IL-4–treated mice (Supplemental Fig. 1D). Given the protection from *L. major* pathology and concurrent reduction in Ag-experienced CD8^+^ T cells observed in anti–IL-4–treated groups, we propose that IL-4 may in part promote *L. major* pathology by promoting activation of CD8^+^ T cells. In addition to T cells, we also examined CD19^+^MHCII^+^ B cells and CD11c^+^MHCII^+^ dendritic cells (DC) in PLN, which were similar in PBS- and anti–IL-4–treated groups (Supplemental Fig. 2).

### *Chronic IL-4 neutralization inhibits optimal cytokine production by both CD4*^+^ *and CD8*^*+*^ *T cells during* L. major *infection*

**FIGURE 2.**
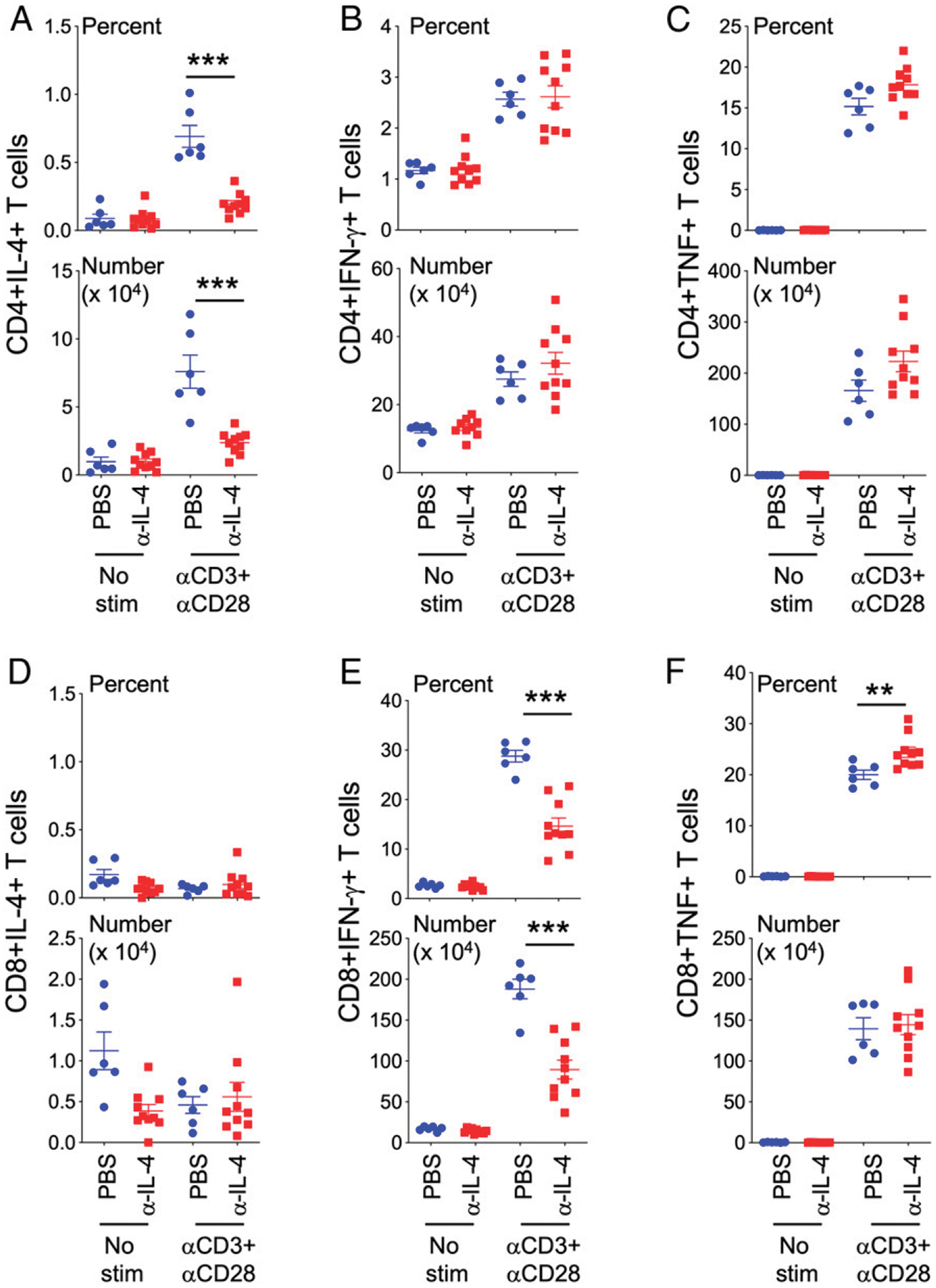
IL-4 is not only required for subsequent IL-4 production by CD4^+^ T cells, but also for optimal IFN-γ and TNF production by CD8^+^ T cells. PBS- or anti-IL-4–treated mice infected with *L. major* were euthanized on day 32. PLN cells were stimulated with or without anti-CD3 and anti-CD28 Abs for 4 h in the presence of brefeldin A. T cells and cytokine production were evaluated by flow cytometry. Percentage and number of CD4^+^ T cells producing IL-4 (**A**), IFN-γ (**B**), and TNF (**C**), and CD8^+^ T cells producing IL-4 (**D**), IFN-γ (**E**), and TNF (**F**). Each dot represents an individual mouse. Data represent the mean ± SEM. **p < 0.01, ***p < 0.001.

A Th1 versus Th2 immune response during *L. major* infection determines the course of the infection (1). To study T cell responses, PLN cells were restimulated with anti-CD3 and anti-CD28, and Th1 (IFN-γ) and Th2 (IL-4) cytokine production by CD4^+^ and CD8^+^ T cells was assessed by flow cytometry (Fig. 2, Supplemental Fig. 3). IL-4 signals in a positive feedback loop to promote IL-4 production by CD4^+^ T cells. Thus, IL-4–producing CD4^+^ T cells (both frequency and numbers) were significantly blunted in the anti–IL-4–treated BALB/c mice during *L. major* infection (Fig. 2A, Supplemental Fig. 3A). Indeed, the levels of IL-4 produced by CD4^+^ T cells in the anti–IL-4–treated groups were barely above background levels (Fig. 2A, Supplemental Fig. 3A). Interestingly, we did not observe any increase in IFN-γ–producing CD4^+^ T cells, a hallmark for Th1 CD4^+^ T cells, in the protected anti–IL-4–treated groups (Fig. 2B, Supplemental Fig. 3A). Frequency and numbers of TNF-producing CD4^+^ T cells trended higher in the anti–IL-4–treated groups compared with PBS-treated groups (Fig. 2C, Supplemental Fig. 3A). Thus, the protection observed in IL-4–neutralized groups does not correlate with increased IFN-γ production by CD4^+^ T cells.

In addition to CD4^+^ T cells, we also assessed the impact of chronic IL-4 neutralization on CD8^+^ T cell responses. As expected, CD8^+^ T cells do not produce IL-4 above background levels (Fig. 2D, Supplemental Fig. 3B). However, IFN-γ–producing CD8^+^ T cell frequency and numbers were considerably reduced in the anti–IL-4–treated groups (Fig. 2E, Supplemental Fig. 3B). Given that Ag-experienced CD8^+^CD11a^hi^ T cells were also reduced (Supplemental Fig. 1D), we reasoned that CD8^+^ T cell responses were defective in *L. major*–infected IL-4–neutralized BALB/c mice. However, the ability of these CD8^+^ T cells to produce TNF remained unaffected (Fig. 2F, Supplemental Fig. 3B). Moreover, frequencies of TNF-producing CD8^+^ T cells were significantly increased in the anti–IL-4–treated groups, suggesting a negative role for IL-4 in limiting TNF production by T cells (Fig. 2F, Supplemental Fig. 3B). Altogether, these studies suggest that IL-4 produced during *L. major* infection of BALB/c mice not only primed Th2 CD4^+^ T cell responses but also instructed IFN-γ–producing CD8^+^ T cells.

### *Acute IL-4 neutralization is sufficient to provide protection from* L. major *infection in BALB/c mice*

**FIGURE 3.**
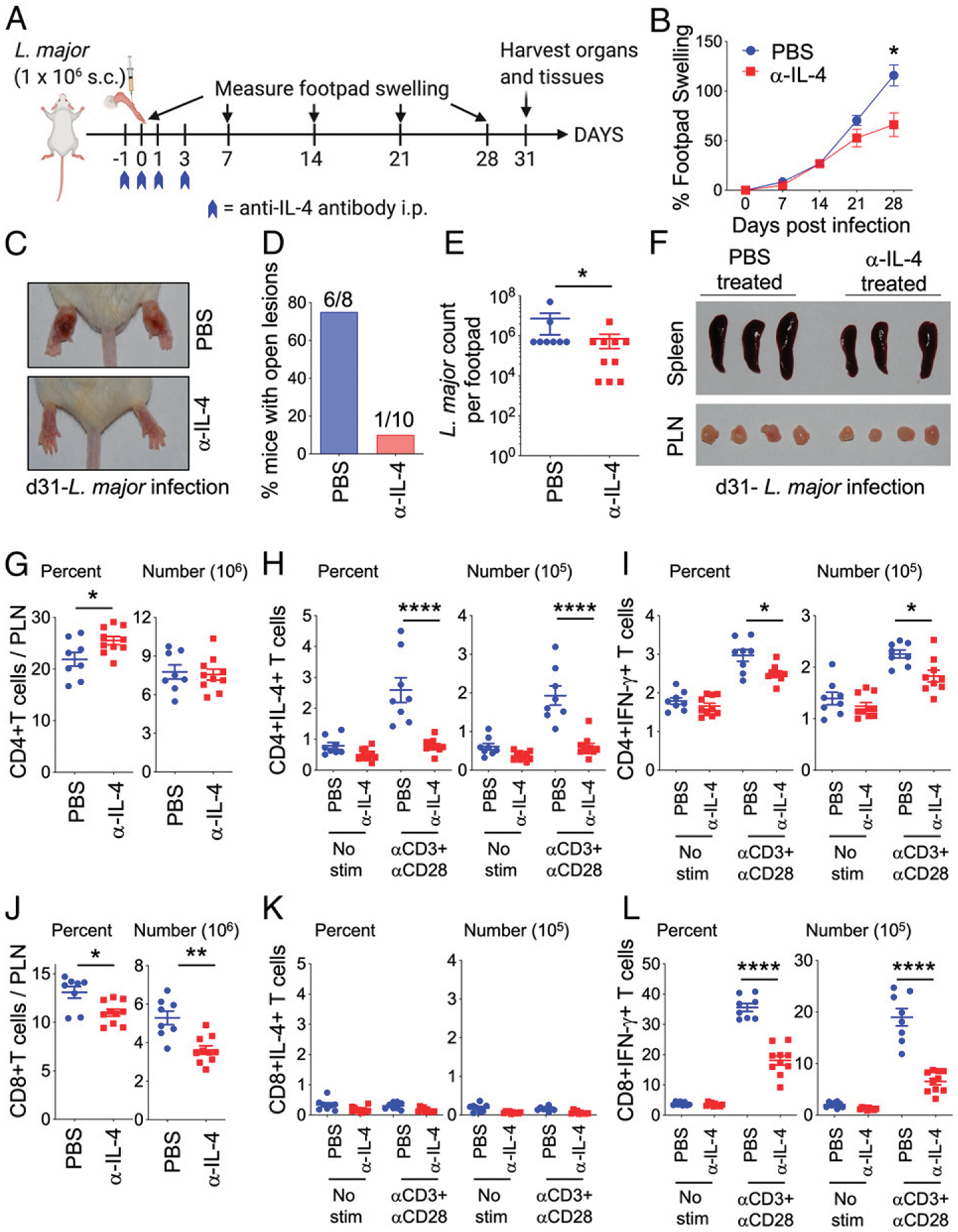
IL-4 neutralization at early timepoints following *L. major* infection of BALB/c mice is sufficient to provide protection and promote protective CD4^+^ and CD8^+^ T cell responses. (**A**) Schematic diagram of experimental design. Mice were infected in the footpads s.c. with 1 million *L. major* metacyclic promastigotes in 50 μl volume. On days −1, 0, 1, and 3 postinfection, mice were injected i.p. with PBS or 400 μg of anti–IL-4 Ab. Footpad measurements were taken once weekly, and mice were euthanized on day 31 postinfection. (**B**) Measurements of footpad swelling in PBS- and anti–IL-4–treated BALB/c mice following *L. major* infection. (**C**) Representative images of footpads of PBS- and anti–IL-4–treated *L. major*–infected mice. (**D**) Graphical representation of lesions in PBS- and anti–IL-4–treated *L. major*–infected mice. (**E**) *L. major* titers in footpads on day 31 postinfection determined by limiting dilution assay. (**F**) Representative images of spleens and PLN from PBS- and anti–IL-4–treated *L. major*–infected mice. (**G**–**L**) PBS- and anti–IL-4–treated mice infected with *L. major* were euthanized on day 31. PLN cells were stimulated with anti-CD3 and anti-CD28 Abs for 4 h in the presence of brefeldin A. T cell and cytokine production was evaluated by flow cytometry. (G) Frequency and absolute number of CD4^+^ T cells. Total frequencies and numbers of IL-4– (H), and IFN-γ– (I) producing CD4^+^ T cells from PBS- and anti–IL-4–treated *L. major*–infected mice. Similarly, frequencies and absolute numbers of CD8^+^ T cells (J), and CD8^+^ T cells producing IL-4 (K), and IFN-γ (L). Each dot represents an individual mouse. Data represent the mean ± SEM. *p < 0.05, **p < 0.01, ****p < 0.0001.

To understand when IL-4 neutralization is important for modulating T cell immune responses, we redesigned our experiments to neutralize IL-4 during the acute phase [i.e., mice were treated with anti–IL-4 mAbs on days −1, 0, 1, and 3 post-*L. major* infection (Fig. 3A)]. We observed that acute IL-4 neutralization was sufficient to provide significant protection from footpad swelling (Fig. 3B). Representative footpad images show that acute IL-4 neutralization attenuated footpad swelling and lesion development in *L. major*–infected BALB/c mice (Fig. 3C). Although 80% of the PBS-treated groups had lesions with open wounds, only 10% of the acute anti–IL-4–treated groups presented with open wound lesions (Fig. 3D). The protection from *L. major* swelling and lesion pathology correlated with significant reduction in *L. major* parasite burden in the acute anti–IL-4–treated groups (Fig. 3E). Representative spleen and PLN from the PBS- and acute anti–IL-4–treated BALB/c mice showed no obvious differences (Fig. 3F). Analysis of the PLN immune cells showed that there was significantly increased frequency of CD4^+^ T cells in the acute anti–IL-4–treated mice, although the numbers remained unchanged (Fig. 3G). Similar to chronic IL-4–neutralized groups, frequency and numbers of CD8^+^ T cells were significantly reduced in the acute IL-4–neutralized groups (Fig. 3J). Ag-experienced CD4^+^CD11a^hi^ T cells remain unchanged; however, Ag-experienced CD8^+^CD11a^hi^ cells were significantly reduced in the acute anti–IL-4–treated groups (Supplemental Fig. 4A, 4B). CD19^+^MHCII^+^ B cells were similar, but CD19^+^MHCII^+^ DC were significantly reduced in the acute anti–IL-4–treated groups (Supplemental Fig. 4C, 4D).

To examine the effect of acute IL-4 neutralization on T cell responses, PLN cells harvested on day 31 post-*L. major* infection were restimulated with anti-CD3 and anti-CD28 for 4 h in brefeldin A, followed by intracellular staining for Th1 and Th2 cytokines. IL-4 production by CD4^+^ T cells was significantly dampened in the acute IL-4–neutralized groups, demonstrating the importance of acute IL-4 in priming Th2 responses (Fig. 3H). Similar to chronic IL-4 neutralization (Fig. 2B), acute IL-4 neutralization did not enhance IFN-γ–producing CD4^+^ T cells. Moreover, in these settings, acute IL-4 neutralization significantly reduced CD4^+^IFN-γ^+^ T cell frequency and numbers (Fig. 3I). In contrast, frequency and numbers of CD4^+^TNF^+^ T cells in PLN were significantly increased in the acute anti–IL-4–treated mice (Supplemental Fig. 4E). As expected, CD8^+^ T cells did not produce IL-4 above background levels (Fig. 3K), but CD8^+^IFN-γ^+^ T cell frequency and numbers were significantly reduced in the acute anti–IL-4–treated mice (Fig. 3L), whereas CD8^+^TNF^+^ T cells were significantly increased (Supplemental Fig. 4F). Altogether, these studies demonstrate that acute IL-4 treatment that neutralized IL-4 over the first 3 d of *L. major* infection was sufficient to inhibit optimal IL-4 and IFN-γ production by CD4^+^ T cells measured on day 31 postinfection. Strikingly, acute neutralization of IL-4 had an even more dramatic effect on attenuating IFN-γ–producing CD8^+^ T cells.

### *Prophylactic anti–IL-4 treatment of BALB/c mice on day 1 provides significant protection from* L. major *infection*

**FIGURE 4.**
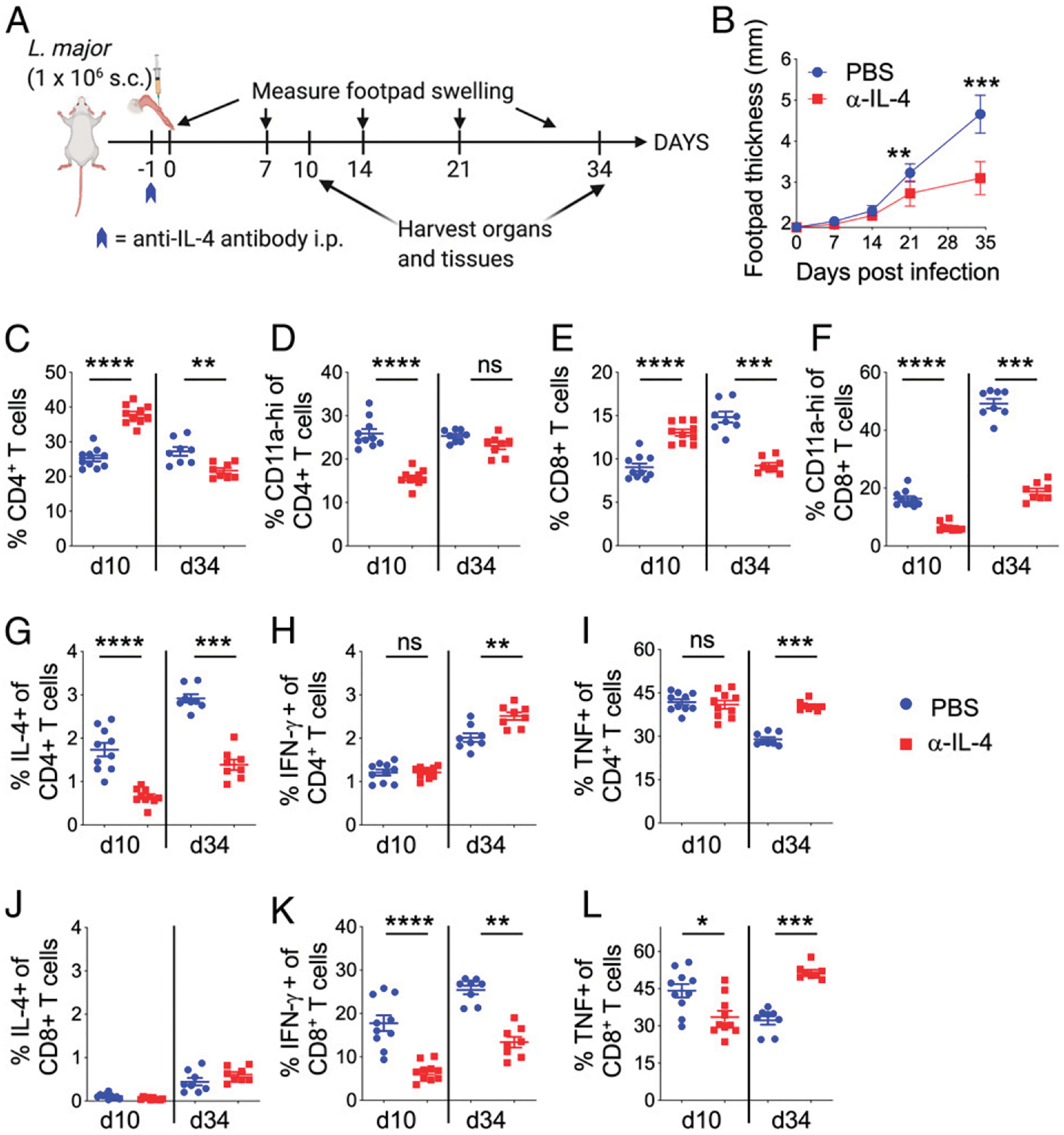
Single dose of prophylactic IL-4 neutralization confers resistance to *L. major* infection in BALB/C mice. (**A**) Schematic diagram of experimental design. Mice were infected in the footpads s.c. with 1 million *L. major* metacyclic promastigotes in 50 μl volume. On day −1, mice were injected i.p. with PBS or 400 μg of anti–IL-4 Ab. Footpad measurements were taken once weekly, and mice were euthanized on days 10 and 31 postinfection. (**B**) Measurements of footpad swelling in PBS- and anti–IL-4–treated BALB/c mice following *L. major* infection. (**C**–**L**) PBS- and anti–IL-4–treated mice infected with *L. major* were euthanized on days 10 and 34. PLN cells were surface stained or stimulated with anti-CD3 and anti-CD28 Abs for 4 h in the presence of brefeldin A to determine T cell and cytokine production. Frequency of CD4^+^ (C), CD4^+^CD11a^hi^ (D), CD8^+^ (E), and CD8^+^CD11a^hi^ (F) T cells in PLN on days 10 and 34 postinfection. ICS staining of anti-CD3– and anti-CD28–stimulated PLN cells for determining frequencies of IL-4– (G), IFN-γ– (H), and TNF- (I) producing CD4^+^ T cells. Similar frequencies of IL-4– (J), IFN-γ– (K), and TNF-producing CD8^+^ T cells (L) in PLN on days 10 and 34 postinfection. Each dot represents an individual mouse. Data represent the mean ± SEM. *p < 0.05, **p < 0.01, ***p < 0.001, ****p < 0.0001.

Our data so far has shown that either chronic (throughout the study length, Fig. 1A) or acute (first 3 days, Fig. 3A) IL-4 neutralization is sufficient to provide protection from *L. major* infection in susceptible BALB/c mice. Importantly, both of these neutralization strategies impeded IL-4 production by CD4^+^ T cells and IFN-γ production by CD8^+^ T cells. To further pinpoint the temporal effect of acute IL-4, we sought to neutralize IL-4 for a very short time. Thus, BALB/c mice were injected with one dose of anti–IL-4 Ab prophylactically, 1 d before *L. major* infection (Fig. 4A). In addition to examining immune responses at the end of the experiment on day 34, we also examined immune responses at an acute time point, on day 10 post-*L. major* infection (Fig. 4A). As shown previously, one single prophylactic dose of anti–IL-4 Ab was sufficient to ameliorate *L. major*–induced footpad swelling (Fig. 4B). The frequency of CD4^+^ T cells in PLN of anti–IL-4–treated mice was significantly increased on day 10 but reversed on day 34 (Fig. 4C). Although the frequency of CD4^+^CD11a^hi^ T cells was similar on day 34 (similar to chronic and acute IL-4 neutralization strategies), on day 10, the frequency of CD4^+^CD11a^hi^ T cells was significantly reduced in the anti–IL-4–treated mice (Fig. 4D). In agreement with chronic and acute IL-4 neutralization, prophylactic IL-4 neutralization resulted in significantly reduced CD8^+^ T cell frequency on day 34 (Fig. 4E). Interestingly, these CD8^+^ T cell frequencies were significantly increased on day 10 postinfection in anti–IL-4–treated mice (Fig. 4E). Despite differences in CD8^+^ T cell frequency at acute and chronic timepoints following *L. major* infection (Fig. 4E), Ag-experienced CD8^+^CD11a^hi^ T cell frequency was significantly reduced on both days 10 and 34 postinfection (Fig. 4F).

Intracellular cytokine secretion following restimulation of lymph node cells with anti-CD3 and anti-CD28 showed that CD4^+^ T cells from acute IL-4–treated mice produced significantly reduced levels of IL-4 on both days 10 and day 34 post-*L. major* infection (Fig. 4G). Interestingly, in these prophylactically IL- 4–neutralized mice, IFN-γ– and TNF-producing CD4^+^ cells were similar on day 10 but significantly increased on day 34 (Fig. 4H, 4I). As expected, CD8^+^ T cells do not produce significant levels of IL-4 (Fig. 4J). IFN-γ producing CD8^+^ T cells were significantly reduced on both days 10 and 34post-*L. major* infection in anti–IL-4–treated mice (Fig. 4K). Interestingly, TNF-producing CD8^+^ T cells were significantly reduced on day 10 postinfection but increased on day 34 postinfection in anti–IL-4–treated mice (Fig. 4L).

Altogether, these data suggest that IL-4 produced early during *L. major* infection of BALB/c mice governs T cell responses and the course of disease pathogenesis. Specifically, IL-4 produced during the early timepoints programs Th2 CD4^+^ T cells, which produces more IL-4 in a positive feedback loop. In addition, we showed that IL-4 produced at early timepoints during *L. major* infection of BALB/c mice governs CD8^+^ T cell cytokine responses and is required for optimal production of IFN-γ by these CD8^+^ T cells.

### IL-4 neutralization at chronic time points does not change disease course but partially impacts T cell responses

**FIGURE 5.**
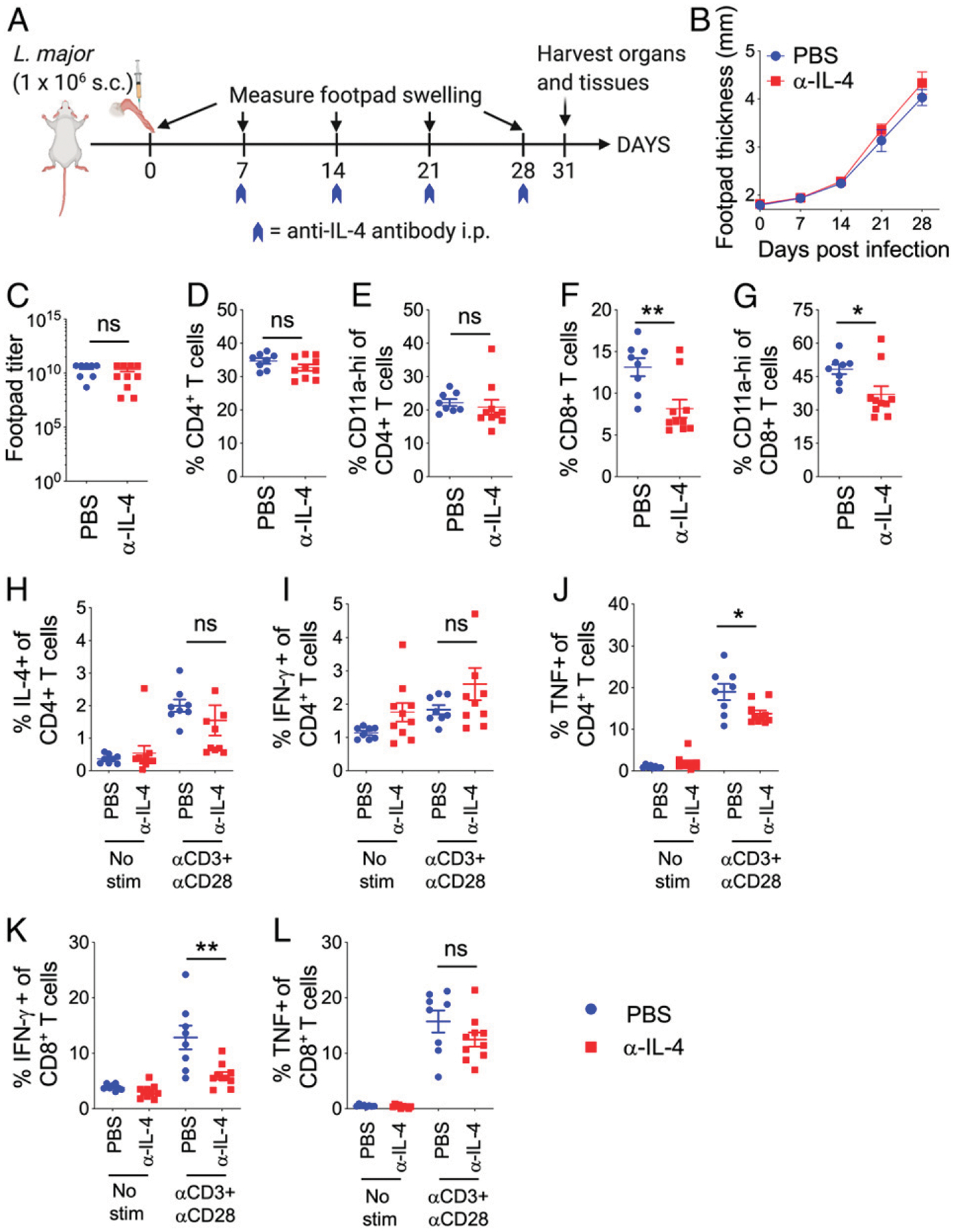
Neutralization of IL-4 after day 7 post*-L. major* infection in BALB/C mice does not provide protection. (**A**) Schematic diagram of experimental design. Mice were infected in the footpads s.c. with 1 million *L. major* metacyclic promastigotes in 50 μl volume. On days 7, 14, 21, and 28, mice were injected i.p. with PBS or 400 μg of anti–IL-4 Ab. Footpad measurements were taken once weekly, and mice were euthanized on day 31 postinfection. (**B**) Measurements of footpad swelling in PBS- and anti–IL-4–treated BALB/c mice following *L. major* infection. (**C**) *L. major* titers in footpads on day 31 postinfection determined by limiting dilution assay. (**D**–**L**) PBS- and anti–IL-4–treated mice infected with *L. major* were euthanized on day 31. PLN cells were surface stained or stimulated with anti-CD3 and anti-CD28 Abs for 4 h in the presence of brefeldin A to determine T cell and cytokine production. Frequency of CD4^+^ (D), CD4^+^CD11a^hi^ (E), CD8^+^ (F), and CD8^+^CD11a^hi^ (G) T cells in PLN on day 31 postinfection. ICS staining of anti-CD3– and anti-CD28–stimulated PLN cells for determining frequencies of IL-4– (H), IFN-γ– (I) and TNF- (J) producing CD4^+^ T cells. Similar frequency of IFN-γ– (K) and TNF- (L) producing CD8^+^ T cells in the PLN on day 31 postinfection. Each dot represents an individual mouse. Data represent the mean ± SEM. *p < 0.05, **p < 0.01.

To address the role of IL-4 produced at later timepoints in disease course and in modulating T cell responses, we treated *L. major*–infected BALB/c mice with anti–IL-4 Ab on days 7, 14, 21, and 28 postinfection (Fig. 5A). In contrast to acute neutralization of IL-4, neutralization of IL-4 produced at later timepoints did not affect disease course as measured by footpad swelling and *L. major* burden (Fig. 5B, 5C). Neutralization of IL-4 at these later timepoints did not impact CD4^+^ T cell frequency or their activation measured by CD11a expression (Fig. 5D, 5E). However, neutralization of IL-4 at these chronic timepoints still led to reduced frequency of CD8^+^ T cells and Ag-activated CD8^+^CD11a^hi^ T cells (Fig. 5F, 5G). These results are indeed similar to what was observed with acute IL-4 neutralization (Fig. 4, Supplemental Figs. 1, 4), and suggest that both early and late IL-4 produced during *L. major* infection governs CD8^+^ T cell activation. Interestingly, we did not find any significant difference in IL-4– or IFN-γ–producing CD4^+^ T cells in IL-4–neutralized mice compared with controls (Fig. 5H, 5I). Frequency of CD4^+^TNF^+^ cells was slightly reduced in IL-4–neutralized groups (Fig. 5J). Analysis of intracellular cytokine production by CD8^+^ T cells demonstrated that IL-4 produced at later timepoints was partially required for optimal IFN-γ but not TNF production by CD8^+^ T cells (Fig. 5K, 5L). Altogether, these studies demonstrate that, whereas IL-4 produced at chronic timepoints (day 7 postinfection and later) did not affect the disease course or CD4^+^ Th2 responses, chronically produced IL-4 were partially required for optimal IFN-γ production by CD8^+^ T cells.

### Direct IL-4 signaling on T cells regulates Th1 and Th2 programming via upregulation of concomitant transcription factors

To examine the impact of IL-4 signaling on the generation of Th1 and Th2 immune responses, we stimulated whole splenocytes with anti-CD3 in the presence or absence of rIL-4. Flow cytometric analysis showed that anti-CD3 stimulation of whole splenocytes increased levels of GATA3 (Th2 transcription factor) in CD4^+^ T cells, which was further enhanced in the presence of rIL-4 (Supplemental Fig. 5A). Interestingly, rIL-4 also enhanced Tbet (Th1 transcription factor) expression by CD4^+^ T cells. In addition, rIL-4 significantly increased CD8 expression on CD8^+^ Tcells (Supplemental Fig. 5B). Although CD8^+^T cells do not produce IL-4 above background levels (as demonstrated by flow cytometry), anti-CD3 stimulation of whole splenocytes induced robust GATA3 expression by CD8^+^ T cells that was further enhanced by rIL-4 (Supplemental Fig. 5B). Furthermore, rIL-4 enhanced aCD3-induced Tbet expression in CD8^+^ T cells (Supplemental Fig. 5B).

**FIGURE 6.**
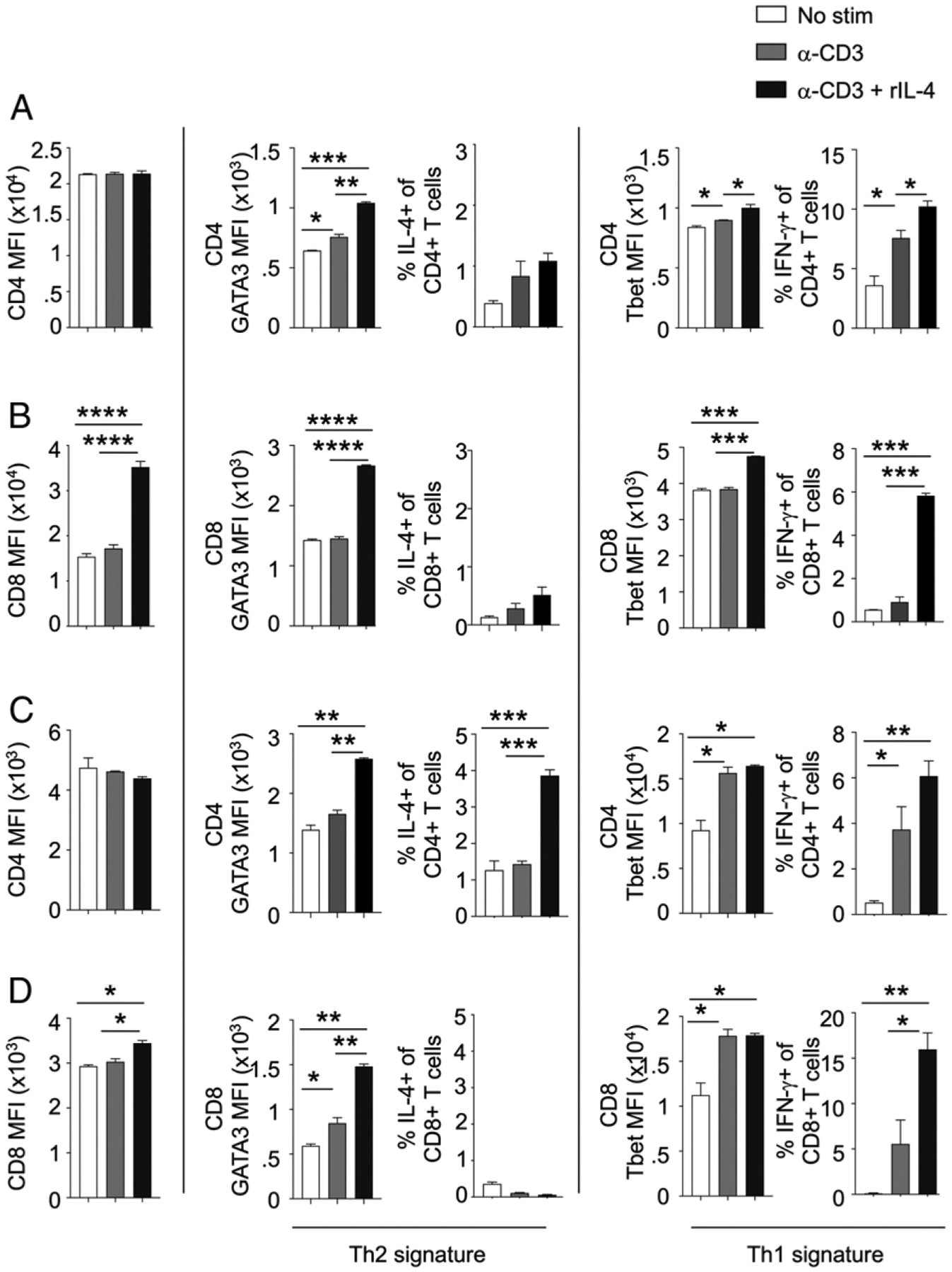
rIL-4 enhances anti-CD3–mediated upregulation of Th1 and Th2 transcription factors and cytokines in purified CD4^+^ and CD8^+^ T cells in vitro. (**A** and **B**) Following isolation, magnetically purified CD4^+^ and CD8^+^ T cells were stimulated with αCD3 in the presence or absence of rIL-4 for 24 h. For intracellular staining of cytokines, brefeldin A was added for 4 h, and cells were processed for flow cytometry analysis. (A) Isolated CD4^+^ T cells were analyzed by flow cytometry for CD4 MFI, GATA3 MFI, percentage IL-4 of CD4^+^ T cells, Tbet MFI, and percentage IFN-γ of CD4^+^ T cells. (B) Isolated CD8^+^ T cells were analyzed by flow cytometry for CD8 MFI, GATA3 MFI, percentage IL-4 of CD8^+^ T cells, Tbet MFI, and percentage IFN-γ of CD8^+^ T cells. (**C** and **D**) PBMCs isolated from human blood were stimulated with αCD3 in the presence or absence of rhIL-4 for 48 h. CD4^+^ and CD8^+^ T cells were analyzed for expression of Th1 and Th2 factors. (C) Expression of CD4 MFI, GATA3 MFI, percentage IL-4 of CD4^+^ T cells, Tbet MFI, and percentage IFN-γ of CD4^+^ T cells in CD4^+^ T cells from PBMC. (D) Expression of CD8 MFI, GATA3 MFI, percentage IL-4 of CD8^+^ T cells, Tbet MFI, and percentage IFN-γ of CD8^+^ T cells in CD8^+^ T cells from PBMC. *n* = 3 mice per group. Experiments are representative of at least three independent experiments. Data represent the mean ± SEM. *p < 0.05, **p < 0.01, ***p < 0.001, ****p < 0.0001.

To further examine whether IL-4 signaling directly on T cells was sufficient to program the Th1 and Th2 responses observed in whole splenocytes, we isolated CD4^+^ and CD8^+^ T cells that were subsequently stimulated with anti-CD3 in the presence or absence of rIL-4. The levels of CD4 coreceptor on CD4^+^ T cells remained unchanged with anti-CD3 or anti-CD3 plus rIL-4 stimulation (Fig. 6A). As observed with whole splenocyte stimulation, anti-CD3 stimulation of purified CD4^+^ T cells upregulated GATA3 expression and frequency of IL-4^+^CD4^+^ T cells, both of which were further enhanced in the presence of rIL-4 (Fig. 6A, Supplemental Fig. 6A). In addition to promoting a Th2 signature, rIL-4 also enhanced aCD3-induced Tbet expression and frequency of IFN-γ^+^CD4^+^ T cells in CD4^+^ T cells (Fig. 6A, Supplemental Fig. 6A). Similar to whole splenocyte stimulation, rIL-4 addition to purified CD8^+^ T cells induced robust upregulation of surface CD8 coreceptor levels (Fig. 6B, Supplemental Fig. 6B). In purified CD8^+^ T cells, anti-CD3 stimulation did not induce any significant levels of GATA3; however, anti-CD3 plus rIL-4 stimulation resulted in significant upregulation of GATA3 (Fig. 6B, Supplemental Fig. 6B). As expected, we did not detect any significant levels of IL-4 in control or stimulated CD8^+^ T cells (Fig. 6B, Supplemental Fig. 6B). In concurrence with the in vivo data, the addition of rIL-4 during anti-CD3 stimulation of purified CD8^+^ T cells led to significantly increased Tbet expression, which also correlated with significantly increased frequency of IFN-γ^+^CD8^+^ T cells (Fig. 6B, Supplemental Fig. 6B). Similar enhancement of Th1 and Th2 programs were also observed in purified CD8^+^ T cells stimulated with PMA and ionomycin in the presence of rIL-4 (Supplemental Fig. 7). Altogether, these data suggest that rIL-4 signaling directly on CD4^+^ and CD8^+^ T cells is sufficient to enhance both Th1 and Th2 programs.

Finally, to test whether the observed role of rIL-4 can also be observed in human T cells, we stimulated PBMCs with anti-CD3 in the presence of recombinant human(rh) IL-4 (Fig. 6C, 6D, Supplemental Fig. 6C, 6D). Similar to the murine cells, rhIL-4 enhanced both Th1 (increased Tbet mean fluorescence intensity [MFI] and increased IFN-γ^+^ T cell frequency) and Th2 signatures (increased GATA3 MFI and increased IL-4^+^ T cell frequency) in CD4^+^ and CD8^+^ T cells from anti-CD3 stimulated PBMC (Fig. 6C, 6D, Supplemental Fig. 6C, 6D).

**FIGURE 7.**
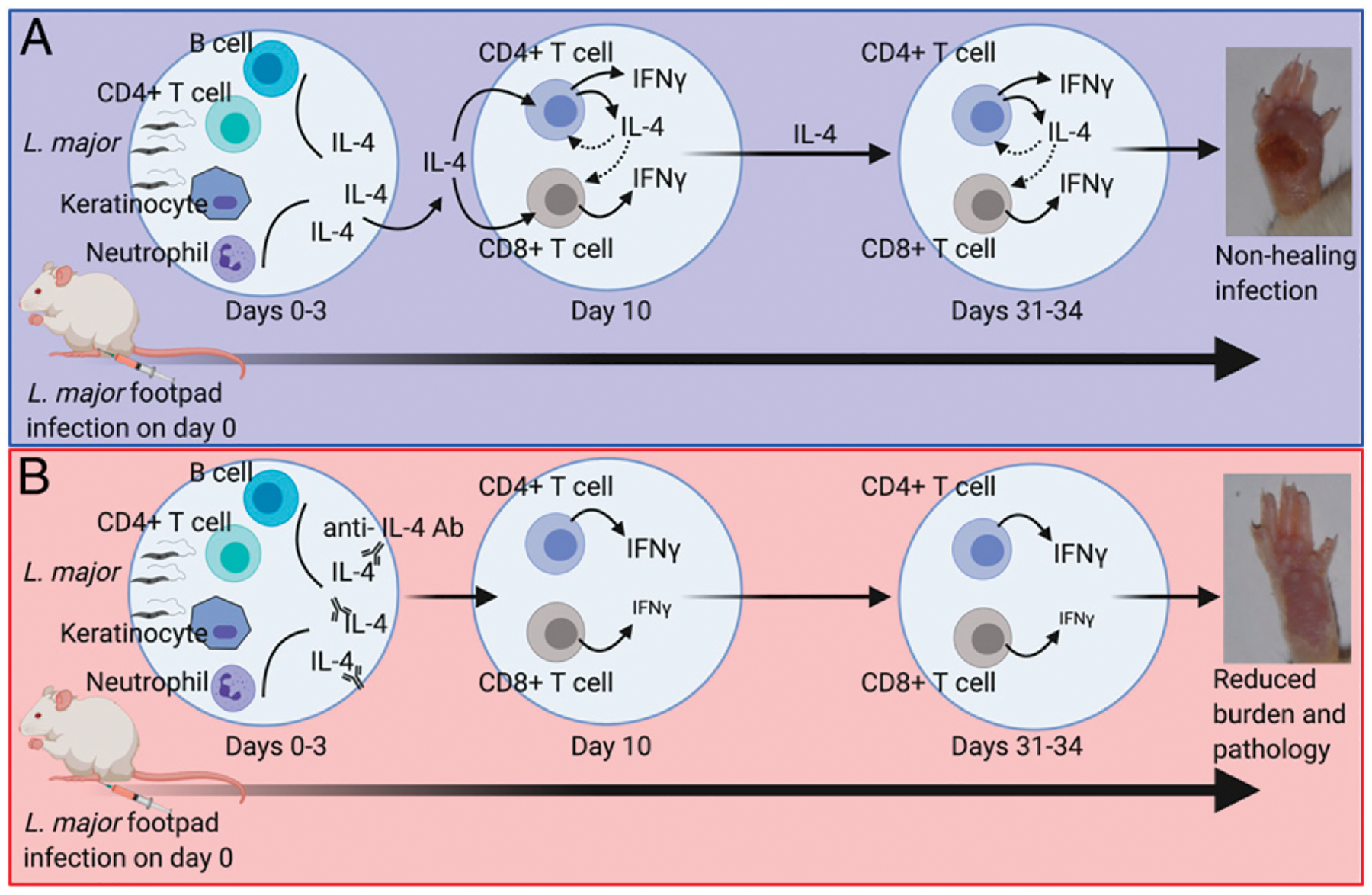
Proposed model of IL-4–mediated pathogenesis during *L. major* infection in BALB/c mice. (**A**) *L. major* infection of BALB/c mice promotes a nonhealing infection. (**B**) Neutralization of IL-4 during the early time points following *L. major* infection of BALB/c mice results in complete abrogation of IL-4–producing CD4^+^ T cells and reduction of IFN-γ production by CD8^+^ T cells. Together, these T cell responses promote successful clearance of *L. major* parasites and amelioration of immunopathology.

Taken together, our data demonstrate that IL-4 can directly engage CD4^+^ and CD8^+^ T cells to promote both Th1 and Th2 responses (Fig. 7).

## DISCUSSION

IL-4 neutralization (via anti–IL-4 Ab) results in resistance to *Leishmania* spp., demonstrating a pathogenic role for IL-4 during infection (14). Indeed, IL-4 neutralization has been shown to decrease Th2 responses and increase Th1 responses, supporting a role for IL-4 as a hallmark Th2 cytokine (16). In contrast, rIL-4 treatment of BALB/c mice promotes resistance, not susceptibility, to *Leishmania* spp. infection (19). In this study, we analyzed temporal inhibition/neutralization of IL-4 using anti–IL-4 Ab to examine the role of IL-4 in leishmania pathogenesis. As demonstrated previously, we observed that inhibition of IL-4 at early timepoints (days 21–3 postinfection) but not late timepoints (beyond day 7 postinfection) provided significant protection and resistance to *L. major* infection (Fig. 7). Interestingly, analysis of cellular responses revealed that IL-4 neutralization abrogated not only Th2 but Th1 responses as well. Specifically, IL-4 neutralization dramatically reduced the frequency and numbers of IFN-γ–producing CD8^+^ T cells. Mechanistically, we were able to show that direct IL-4 signaling on CD4^+^ and CD8^+^ T cells promotes Th1 and Th2 responses during T cell activation. Thus, our results could potentially explain the discrepant results observed from anti–IL-4–neutralization versus rIL-4–treatment studies. Specifically, in vivo rIL-4 treatment of susceptible mice could promote robust IFN-γ production from both CD4^+^ and CD8^+^ T cells, providing protection from *Leishmania* spp. infection. Indeed, Biedermann et al. (19) observed that in vivo treatment of BALB/c mice with rIL-4 promoted IL-12 production by DC, which subsequently primed a Th1 response and IFN-γ production during *L. major* infection.

What are the potential sources of IL-4 during *L. major* infection? Our studies show that IL-4 produced during the early timepoints following infection plays a central role in programming CD4^+^ and CD8^+^ T cell responses that promote leishmaniasis in BALB/c mice. Previous studieshave shown that CD4^+^ T cells in the lymph nodes express IL-4 rapidly, within the first 16 hours of *L. major* infection (40–42). Specifically, Vβ5-Vα8 CD4^+^ T cells produced IL-4within the first 24 hours, and this early burst of IL-4 was required to promote Th2 responses and susceptibility to *L. major* infection (43). In addition to these CD4^+^ T cells, recent studies have suggested involvement of several other cell types as a potential early source of IL-4. Depletion of neutrophils prior to infection with *L. major* in BALB/c mice prevented an early burst of IL-4 mRNA expression in draining lymph nodes, suggesting a potential role for neutrophils in IL-4 production (44). More recently, keratinocytes have been implicated as a potential source of early IL-4 within the *L. major*–infected footpads in both BALB/c and C57BL/6 mice (45), although production of IL-4 by keratinocytes in C57BL/6 mice has been disputed (46). B cells from BALB/c mice have also been shown to express high levels of IL-4 mRNA on days 1 and 2 post-*L. major* infection, which was dependent on IL-4Ra expression on these cells (47). Although this study demonstrated B cells as a potential early source ofIL-4, these cells might not be the primary source of IL-4 following infection.

In C57BL/6 mice following *L. major* infection, mast cells were found to express IL-4 mRNA during the first 3 days; however, this mRNA expression did not result in the production of functional IL-4 by these cells (46). IL-4 produced at these early timepoints then signals various immune cell populations to promote leishmaniasis. Studies with conditional deletion of IL-4Rα on various immune cell populations have shed light on this particular front. IL-4 signaling to CD4^+^ T cells (48) and B cells (47) via IL-4Rα promotes *L. major* pathogenesis in BALB/c mice. In contrast, IL-4 signaling on DC is required to control *L. major* infection in BALB/c mice (49). It is important to note that IL-4Rα deficiency also blocks IL-13 signaling in addition to IL-4. Thus, the resistance or susceptibility observed in mouse models with conditional deletion of IL-4Rα could be partially attributed to IL-13 as well. At chronic timepoints, CD4^+^ T cells are the major producers of IL-4. Interestingly, our data show that blocking this chronic IL-4 has no effect on Th2 programming (i.e., the CD4^+^ T cell’s ability to produce IL-4), suggesting acute IL-4 is critical for exclusively programing a CD4^+^ Th2 fate and that the autocrine IL-4 signaling on CD4^+^ T cells is not important. However, IL-4 produced at these late timepoints (by CD4^+^ T cells) is still required for optimal CD8^+^ T cell activation and production of IFN-γ. These results suggest that, unlike CD4^+^ T cells, both early and late IL-4 are required for optimal programming of CD8^+^ T cells during *L. major* infection.

Our data clearly show that IL-4 neutralization not only abrogates IL-4–producing T cells, but also reduces IFN-γ–producing T cells, especially at chronic timepoints when there are observable differences in *L. major* lesions between controls and IL-4–treated BALB/c mice. This is in contrast to several prior studies suggesting that IL-4 neutralization skews Th2 responses to Th1, thus providing protection from *L. major* infection. Instead, we demonstrate that, in addition to inhibiting IL-4 production and thus a Th2 response, IL-4 neutralization also tempers IFN-γ production, especially by CD8^+^ T cells. Thus, our data demonstrate that the protection observed following IL-4 neutralization is associated with subsequent inhibition of IL-4–producing CD4^+^ T cells, but importantly, the protection is not due to an increase in IFN-γ–producing T cells. In fact, our results demonstrate that, in addition to IL-4–producing T cells, IFN-γ–producing T cells are also concomitantly reduced in IL-4–neutralized groups. It should be noted, however, that, whereas IL-4 neutralization completely abrogates IL-4 production, IFN-γ production by T cells is only partially inhibited (especially in CD4^+^ T cells). Based on our data, we posit that, whereas IFN-γ is critical for providing protection against *L. major* infection, too much IFN-γ may actually promote immunopathology. Thus, IL-4 neutralization may provide protection by limiting not only IL-4–producing T cells but also IFN-γ–producing CD8^+^ T cells. In support, recent studies by Novais et al. (33) have shown that IFN-γ specifically produced by CD8^+^ T cells promotes immunopathology during *L. major* infection. Although too much IFN-γ production by CD8^+^ T cells may be pathogenic, it should be noted that two different studies have shown a protective role for CD8^+^ T cells during low-dose *L. major* infection of C57BL/6 mice [i.e., mice deficient in CD8^+^ T cells were unable to resolve *L. major* infection (50, 51)].

In contrast to IL-4 and IFN-γ, IL-4 neutralization led to increased TNF production by both CD4^+^ and CD8^+^ T cells during *L. major* infection. Indeed, TNF in combination with IFN-γ can promote rapid clearance of intracellular *L. major* by macrophages in vitro (52). A subsequent study by Liew et al. (53) showed that rTNF treatment induced IFN-γ production during *L. major* infection. Mice lacking TNF are highly susceptible to both cutaneous (54, 55) and visceral (56) leishmaniasis. In support of a protective role for TNF, mice lacking TNFR I but not TNFR II were highly susceptible to *L. major* infections and developed larger nonhealing lesions compared with C57BL/6 controls (57, 58). Thus, it is possible that the protection provided by IL-4 neutralization against *L. major* could in part be attributed to the increased TNF production by T cells in our study. Kanaly et al. (59) showed that TNF is required for resolution of inflammatory lesions following elimination of parasites, and the lesions fail to regress in the absence of TNFR I. Taken together, IL-4–mediated inhibition of TNF production by T cells during *L. major* infection may be a contributing factor that promotes nonhealing infection in BALB/c mice.

One of the consistent changes that we observed in rIL-4–stimulated CD8^+^ T cells was the significant upregulation of the CD8α coreceptor. Compared with control or only anti-CD3 stimulated CD8^+^ T cells, rIL-4 treatment significantly upregulated CD8α expression in both murine and human CD8^+^ T cells. Interestingly, this effect ofrIL-4wasvery specific to CD8α because rIL-4 did not impact CD4 coreceptor expression on CD4^+^ T cells. CD8 coreceptor tuning has been shown to be important for modulating TCR-mediated CD8^+^ T cell activation [i.e., CD8 quantities on CD8^+^ T cells set the threshold for activation (60, 61)]. More importantly, Park et al. (62) showed that certain common γ-chain cytokines, including IL-4, can promote CD8 coreceptor upregulation on mature CD8^+^ T cells that not only increase T cell reactivity but promote survival. Indeed, anti–IL-4–neutralized groups always presented with reduced frequency and numbers of CD8^+^ T cells in our in vivo experiments. Thus, we posit that during *L. major* infection,IL-4 promotesCD8 coreceptor upregulation on CD8^+^ T cells and lowers the threshold of activation to promote robust activation and survival, which in the chronic stages of the disease may contribute to immunopathology.

IL-4 produced during the early phases of *L. major* infection programs both CD4^+^ and CD8^+^ T cell responses. Specifically, this early IL-4 is required to program IL-4–producing Th2 CD4^+^ T cells. Interestingly, early IL-4 did not impede IFN-γ production by CD4^+^ or CD8^+^ T cells; rather, IL-4 was required for optimal activation and IFN-γ production by CD8^+^ T cells. Previous studies have shown that IL-4 signaling induces a state of IL-12 unresponsiveness in T cells [a cytokine deemed important for Th1 programming and protection during cutaneous leishmaniasis (63)], which could then impede Th1 programming (41, 64). Given that our studies with IL-4 neutralization did not find any defect in IFN-γ responses [similar to study by Morris et al. (65)], we posited that IL-4 may directly regulate T cell responses by enhancing the appropriate Th2 and Th1 transcription factors. In support of direct IL-4 signaling on T cells, Radwanska et al. (48) showed that BALB/c mice with conditional deletion of IL-4Rα in CD4^+^ T cells (and thus unable to directly sense IL-4) were resistant to *L. major* infection. Indeed, direct stimulation of purified CD4^+^ and CD8^+^ T cells with anti-CD3 in the presence of rIL-4 enhanced not only Th2 transcription factor GATA3 but also Th1 transcription factor Tbet. Upregulation of these transcription factors was concomitant with upregulation of both IL-4 and IFN-γ in T cells. Thus, mechanistically, IL-4 produced during the course of *L. major* infection may directly upregulate Th1 and Th2 transcription factors and associated cytokines to promote nonhealing *L. major* infection in BALB/c mice.

In conclusion, using a temporal IL-4 neutralization strategy in BALB/c mice during *L. major* infection, we have uncovered a previously unappreciated role for IL-4 in promoting both Th1 and Th2 responses to disseminate a nonhealing cutaneous infection.

## Supplementary Material

1

## Abbreviations used in this article:

DC: dendritic cell
ICS: intracellular cytoplasmic staining
MFI: mean fluorescence intensity
PLN: popliteal lymph node
rh: recombinant human
