# Supplementary material for: Acute IL-4 Governs Pathogenic T Cell Responses during Leishmania major Infection
Source: Immunohorizons. Author manuscript; Available in PMC 2020 Nov 4. (PMC7640617; doi:10.4049/immunohorizons.2000076)
Supplement: 1 [file NIHMS1641858-supplement-1.pdf]

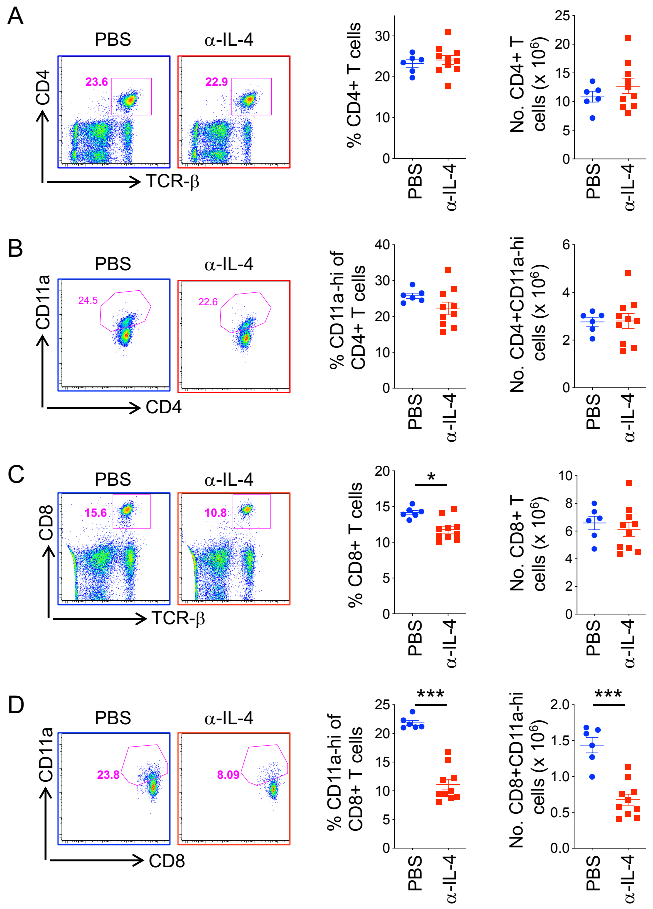

Supplemental Figure 1. IL-4 is required for the maintenance of optimal CD8+ T cell frequency and activation. PBS- or α-IL4-treated *L. major* infected mice were euthanized on day 32, and draining PLN were processed for flow cytometry to detect CD4+ and CD8+ T cells and CD11a expression on the T cells. (A) Representative flow plots, frequency and numbers of CD4+Tcr-β+ T cells in PLN. (B) Representative flow plots, frequency and numbers of CD4+CD11a-hi T cells in PLN. (C) Representative flow plots, frequency and numbers of CD8+Tcr-β+ T cells in PLN. (D) Representative flow plots, frequency and numbers of CD8+CD11a-hi T cells in PLN. Each dot represents an individual mouse. Data represent the mean ± SEM. **P* < 0.05; ****P* < 0.001.

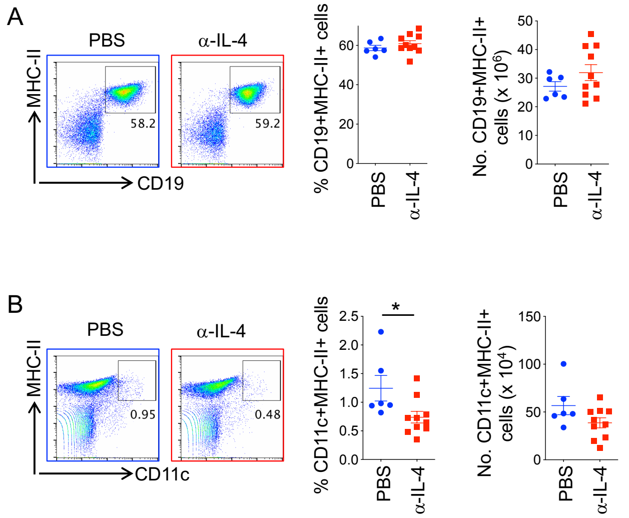

Supplemental Figure 2. Effect of chronic IL-4 neutralization on B cells and dendritic cells (DCs) in the PLN of *L. major*-infected mice. PLN were harvested and stained for CD19+MHCII+ B cells (A) and CD11c+MHCII+ DC (B). Each dot represents an individual mouse. Data represent the mean ± SEM. **P* < 0.05.

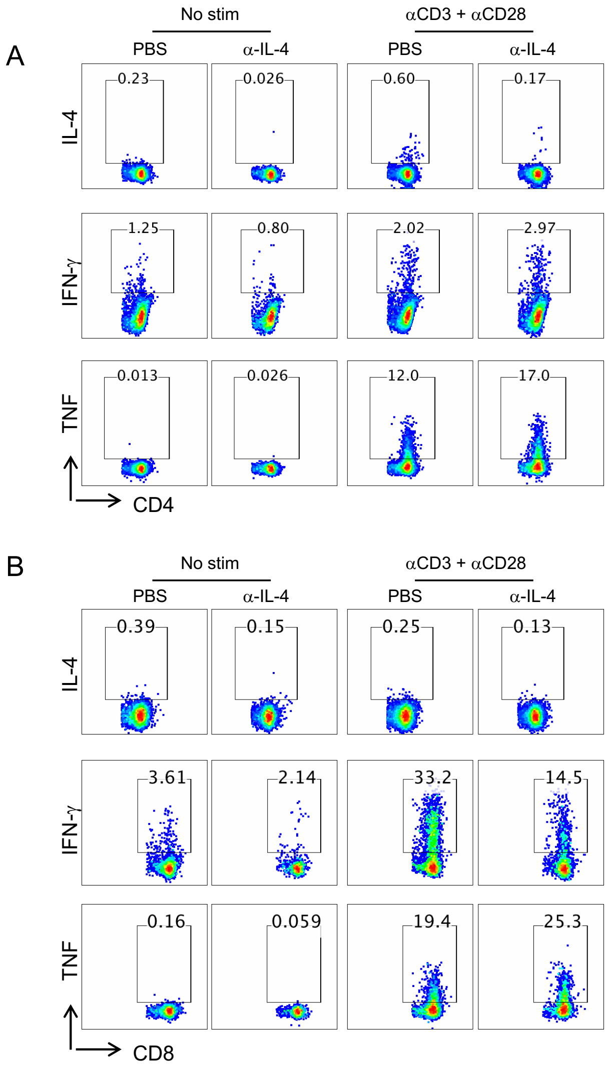

Supplemental Figure 3. Effect of chronic IL-4 neutralization on Th1 and Th2 cytokine production by CD4+ and CD8+ T cells. Related to Fig. 2: PLN were harvested and stimulated with or without anti-CD3 and anti-CD28 antibodies for 4h in the presence of brefeldin A. (A) Representative flow plots of CD4+ T cells producing IL-4, IFN-γ and TNF. (B) Representative flow plots of CD8+ T cells producing IL-4, IFN-γ and TNF.

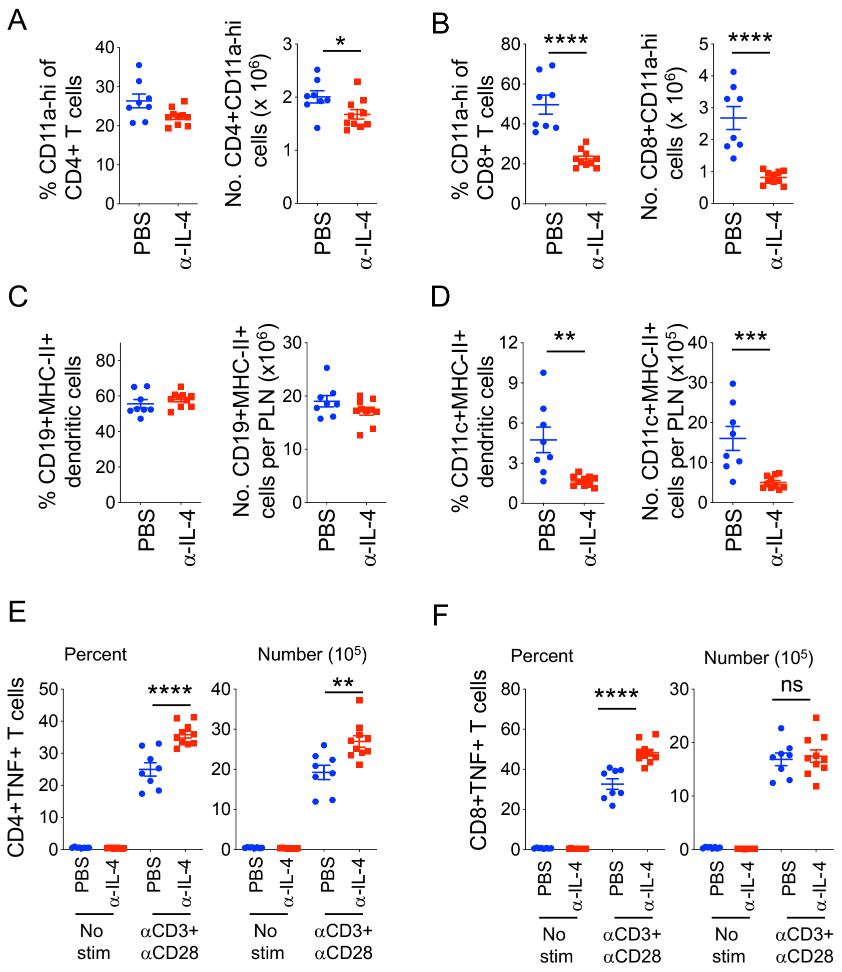

Supplemental Figure 4. Effect of acute IL-4 neutralization on immune cells populations during *L. major* infection. PLN were harvested on day 31 post-infection and the frequency and numbers of activated CD4+CD11a-hi T cells (A), activated CD8+CD11a-hi T cells (B), CD19+MHCII+ B cells (C), CD11c+MHCII+ DC (D) were determined in PBS- vs. a-IL-4-treated mice by surface staining. PLN cells were stimulated with anti-CD3 and anti-CD28 antibodies for 4h in the presence of brefeldin A. TNF producing CD4+ T cells (E), and TNF producing CD8+ T cells (F) were determined by intracellular cytoplasmic staining. Each dot represents an individual mouse. Data represent the mean ± SEM. *$P < 0.05$; **$P < 0.01$; ***$P < 0.001$; ****$P < 0.0001$.

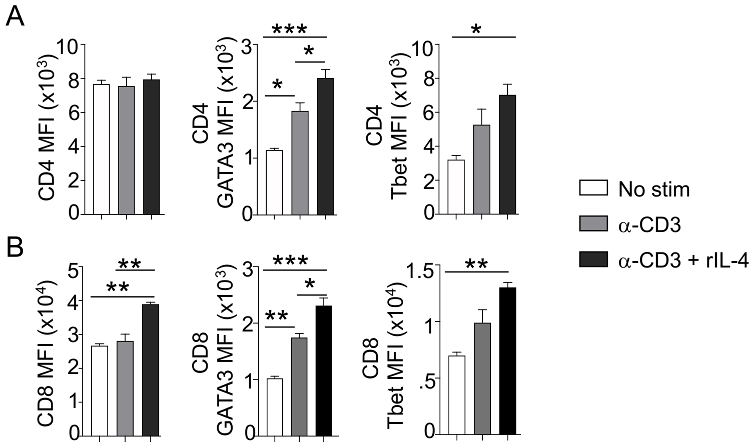

Supplemental Figure 5. rIL-4 promotes anti-CD3 mediated upregulation of Th1 and Th2 transcription factors in CD4+ and CD8+ T cells. Splenocytes from naïve BALB/C mice were stimulated with or without rIL-4 in the presence or absence of anti-CD3 for 24h and cells were processed for flow cytometry analysis. (A) CD4 (left), GATA3 (middle) and Tbet (right) MFI on CD4+ T cells from whole splenocytes that were left untreated (no stimulation), stimulated with anti-CD3 (αCD3) or αCD3+rIL-4 for 24h. (B) CD8 (left), GATA3 (middle) and Tbet (right) MFI on CD8+ T cells from whole splenocytes that were left untreated (no stimulation), stimulated with αCD3 or αCD3+rIL-4 for 24h. Data represent the mean ± SEM. N=3/group. Experiments are representative of at least three independent experiments. *$P < 0.05$; **$P < 0.01$; ***$P < 0.001$.

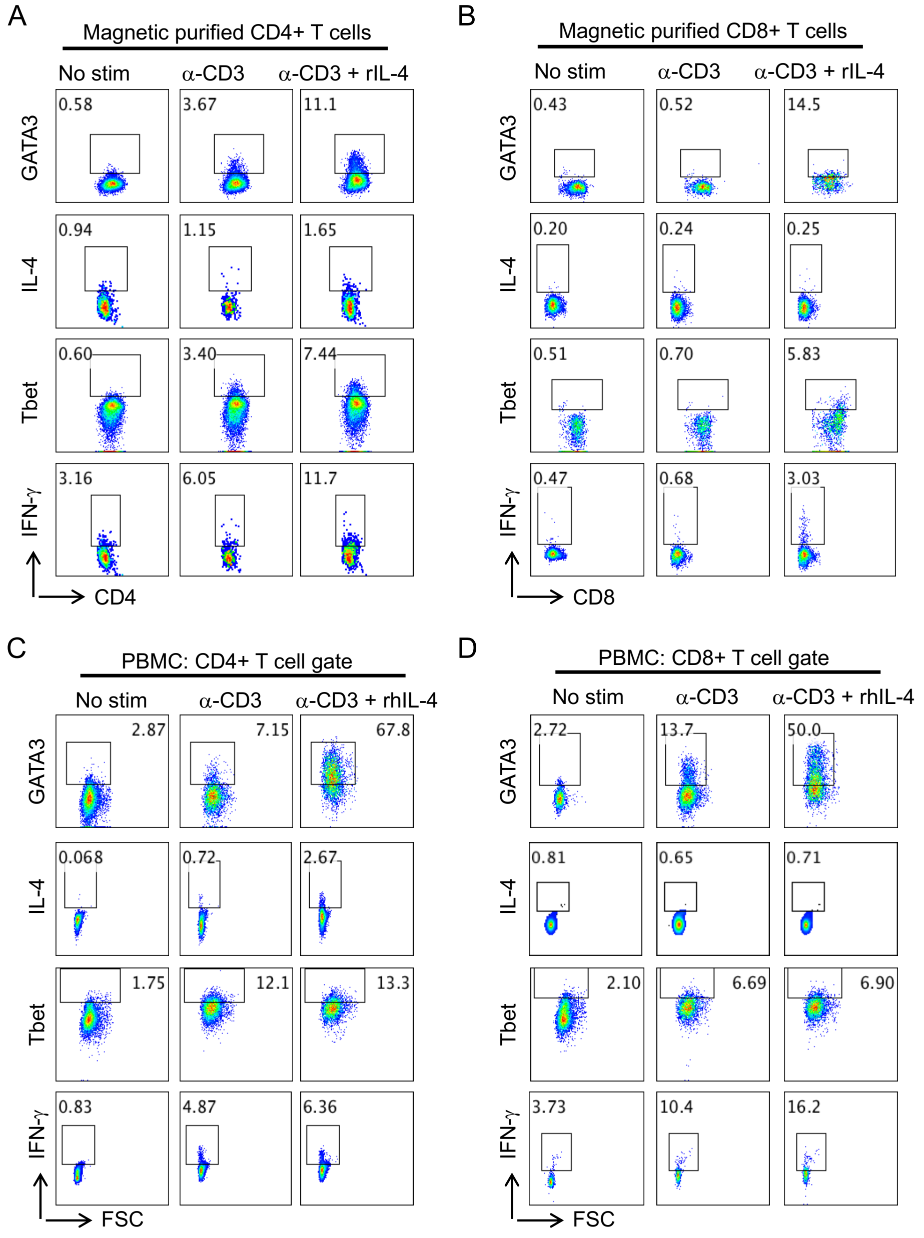

Supplemental Figure 6. rIL-4 promotes anti-CD3 mediated upregulation of Th1 and Th2 transcription factors in purified CD4+ and CD8+ T cells. Related to Fig. 6: (A-B) Magnetically purified CD4+ and CD8+ T cells were stimulated with αCD3 in the presence or absence of rIL-4 for 24h. For intracellular staining of cytokines, brefeldin A was added for 4h and cells were processed for flow cytometry analysis. (A) Representative flow plots depicting frequency of GATA3, IL-4, Tbet and IFN-γ producing CD4+ T cells. (B) Representative flow plots depicting frequency of GATA3, IL-4, Tbet and IFN-γ producing CD8+ T cells. For IL-4 and IFN-γ panels, the x-axis was FSC (C-D) PBMCs from human blood were stimulated with αCD3 in the presence or absence of rhIL-4 for 48h. CD4+ and CD8+ T cells were analyzed for expression of Th1 and Th2 factors. (C) Representative flow plots depicting frequency of GATA3, IL-4, Tbet and IFN-γ producing CD4+ T cells. (D) Representative flow plots depicting frequency of GATA3, IL-4, Tbet and IFN-γ producing CD8+ T cells.

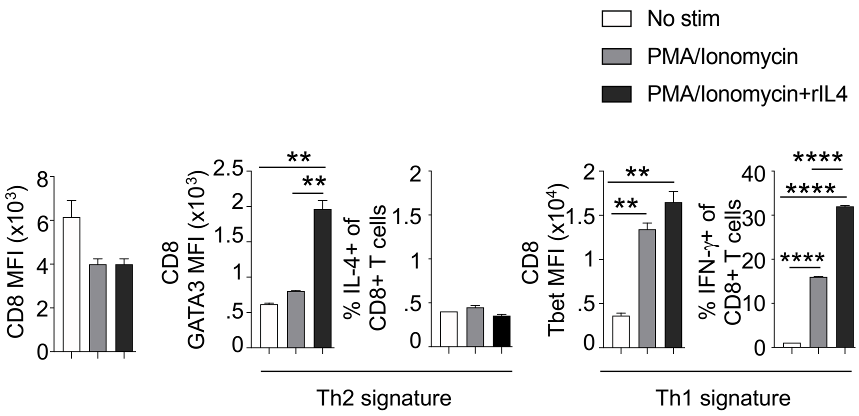

Supplemental Figure 7. rIL-4 enhances PMA and ionomycin induced Th2 and Th1 expression profile on isolated CD8+ T cells. Magnetic-purified CD8+ T cells were treated with PMA/Ionomycin ± rIL-4 for 4h for cytokine analysis in the presence of brefeldin A, or 24h for transcription factors. Stimulated CD8+ T cells were analyzed by flow cytometry analysis to examine Th2 and Th1 signatures as indicated. N = 2 mice per group. Experiments are representative of at least three independent experiments. Data represent the mean ± SEM. $^{**}P < 0.01$; $^{****}P < 0.0001$.
